# Metformin sensitizes sorafenib to inhibit postoperative recurrence and metastasis of hepatocellular carcinoma in orthotopic mouse models

**DOI:** 10.1186/s13045-016-0253-6

**Published:** 2016-03-08

**Authors:** Abin You, Manqing Cao, Zhigui Guo, Bingfeng Zuo, Junrong Gao, Hongyuan Zhou, Huikai Li, Yunlong Cui, Feng Fang, Wei Zhang, Tianqiang Song, Qiang Li, Xiaolin Zhu, Haifang Yin, Huichuan Sun, Ti Zhang

**Affiliations:** Department of Hepatobiliary Surgery, Tianjin Medical University Cancer Institute and Hospital, National Clinical Research Center for Cancer, Key laboratory of Cancer Prevention and Therapy, 24 Bin Shui Road, Hexi District, Tianjin, 300060 People’s Republic of China; Liver Cancer Institute and Zhongshan Hospital, Fudan University, 180 Fenglin Road, Shanghai, 200032 China; Key Laboratory of Carcinogenesis and Cancer Invasion, Ministry of Education, 180 Fenglin Road, Shanghai, 200032 China; Research Center of Basic Medical Science, Tianjin Medical University, Qixiangtai Road, Heping District, Tianjin, 300070 China; Academy of Medical Image, Tianjin Medical University, Tianjin, 300070 People’s Republic of China

**Keywords:** Sorafenib, Metformin, Hypoxia-inducible factors, TIP30, Hepatocellular carcinoma

## Abstract

**Background:**

Sorafenib is recognized as a standard treatment for advanced hepatocellular carcinoma (HCC). However, many patients have to adopt dose reduction or terminate the use of sorafenib because of side effects. In addition, a large number of patients are resistant to sorafenib. Thus, it is essential to investigate the underlying mechanisms of the resistance to sorafenib and seek potential strategy to enhance its efficacy.

**Methods:**

The protein expression of hypoxia-inducible factors (HIF)-2α, 30-kDa HIV Tat-interacting protein (TIP30), E-cadherin, N-cadherin, and pAMPK was detected by Western blot. Cell viability assays were performed to study the influence of metformin and sorafenib on cell proliferation. Annexin V-FITC apoptosis assays were used to detect the influence of metformin and sorafenib on cell apoptosis. The relationship between HIF-2α and TIP30 was studied using gene silencing approach and chromatin immunoprecipitation assay. To investigate the effect of metformin and sorafenib on postoperative recurrence and lung metastasis of HCC in tumor-bearing mice, the mice were orally treated either with metformin or sorafenib once a day for continuous 37 days after the operation to remove the lobe where the tumor was implanted. CD31, Ki67, and TUNEL were examined by immunohistochemistry.

**Results:**

Our study demonstrated that metformin synergized with sorafenib reduced HIF-2α expression as examined by Western blot. Gene silencing approach indicated TIP30 was upregulated after knocking-down of HIF-2α and chromatin immunoprecipitation assay revealed that HIF-2α could bind to TIP30 promoter under hypoxic condition. Cell Counting Kit-8 (CCK8) cell viability assay and Annexin V-FITC apoptosis assay showed that metformin in combination with sorafenib suppressed cell proliferation and promoted cell apoptosis. Besides, combined therapy suppressed epithelial-mesenchymal transition (EMT) process both in vitro and in vivo. Moreover, metformin in combination with sorafenib significantly minimized postoperative recurrence and lung metastasis of HCC in orthotopic mouse model. Combined therapy inhibited CD31 and Ki67 expression but promoted TUNEL expression.

**Conclusions:**

Metformin may potentially enhance the effect of sorafenib to inhibit HCC recurrence and metastasis after liver resection by regulating the expression of HIF-2α and TIP30.

**Electronic supplementary material:**

The online version of this article (doi:10.1186/s13045-016-0253-6) contains supplementary material, which is available to authorized users.

## Background

Hepatocellular carcinoma (HCC) is the fifth most frequently diagnosed cancer in men and the seventh in women worldwide [1]. Surgical resection has been regarded as the main treatment for HCC [2, 3]. However, postoperative recurrence and metastasis are still the main obstacles for long-term survival of HCC [4, 5].

Sorafenib is the first-line oral multi-kinase inhibitor acting on advanced liver cancer. Some evidence suggest that sorafenib can block Raf⁄MEK⁄ERK signaling pathway to inhibit tumor cell proliferation and it can also target the tyrosine kinase receptor vascular endothelial growth factor receptor-2 (VEGFR-2) or platelet-derived growth factor receptor (PDGFR) to produce inhibition of angiogenesis [6–8]. However, sorafenib has been proved to have limited survival benefits with very low response rates because of drug resistance [9]. Hypoxic environment in solid tumor is one of the vital factors for the treatment of resistance [10]. Many evidence indicate that hypoxia-inducible factors (HIFs) are essential for tumor cells to adapt to low oxygen environments [11]. Recent research findings revealed that sorafenib could reduce the expression of HIF-1α, promoting the hypoxic response switch from HIF-1α- to HIF-2α-dependent pathways, resulting in upregulation of HIF-2α [12]. The high expression of HIF-2α induced by sorafenib is the main cause for HCC cells resistant to therapy in hypoxia [13].

It has been reported that, under certain circumstances, anti-angiogenic drugs could heighten invasiveness and increase lymphatic or distant metastasis [14]. Our previous results demonstrated that relatively low dosages of sorafenib promoted HCC invasion and metastasis by downregulating tumor suppressor gene HTATIP2, also known as TIP30, which is a 30-kDa human cellular protein that was purified as a HIV-1 Tat-interacting protein [15]. Until now, the relationship between HIF-2α and TIP30 has yet not been reported.

Metformin is a widely used drug for the treatment of type 2 diabetes [16]. Recently, some articles pointed out that metformin may be associated with reduced cancer incidence in diabetic patients [17, 18], including prostate, breast, pancreas, and liver cancer [19–22]. In addition, it was also reported that metformin could target NLK (Nemo-like kinase) to inhibit non-small cell lung cancer (NSCLC) cell proliferation and stemness [23]. Here, we showed that metformin could increase the sensitivity of HCC cells to sorafenib and inhibit HCC recurrence and metastasis after surgical resection.

## Results

### Metformin-reversed tumor cells resistance to sorafenib in hypoxia

**Fig. 1:**
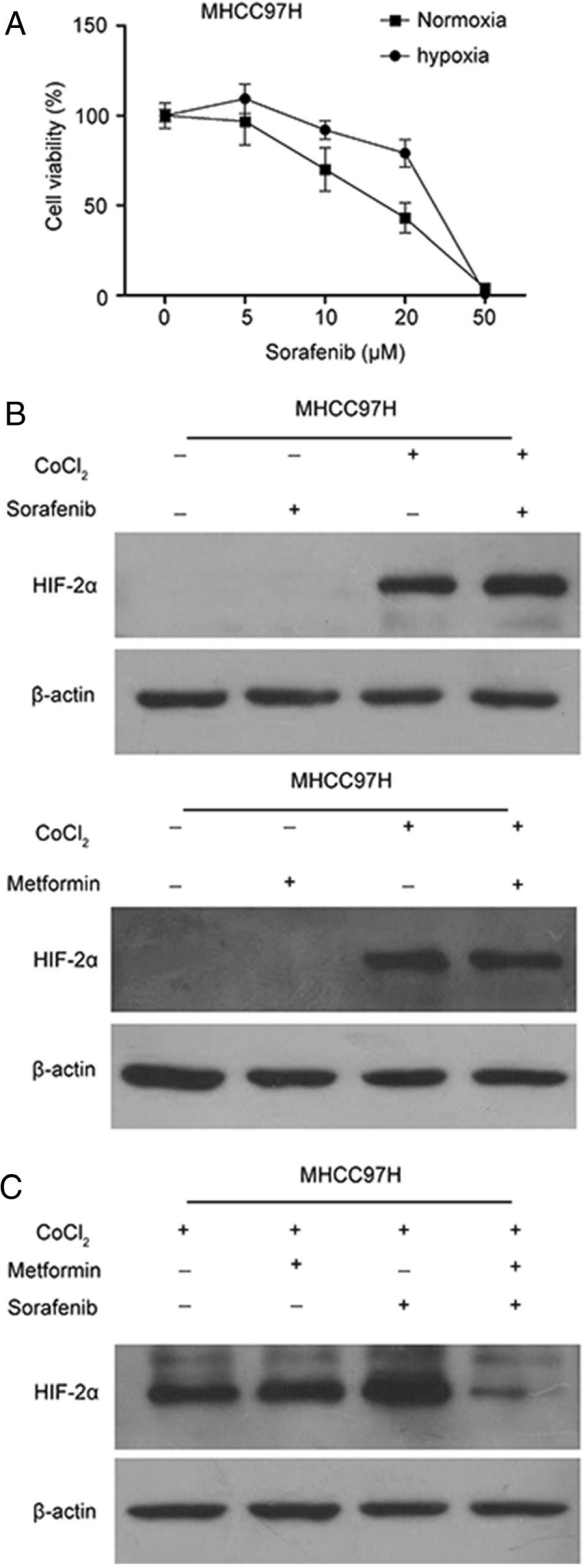
Metformin sensitized hypoxic tumor cells to sorafenib. **a** Hypoxic tumor cells were resistant to sorafenib compared to cells under normoxia. MHCC97H cells were treated with 400 μM CoCl_2_ for 24 h, then incubated with sorafenib or metformin for another 48 h. Cell viability, assessed by the CCK8 method, was expressed as the percent of control for different concentrations. Western blot was used to evaluate the effect of metformin and sorafenib alone or combined on HIF-2α. **b** Under CoCl_2_ (400 μM) treatment, metformin had slight effect on HIF-2α protein expression, but sorafenib promoted HIF-2α protein expression. **c** Combined treatment of metformin and sorafenib noticeably minimized the expression of HIF-2α under CoCl_2_ (400 μM) treatment

MHCC97H cells were incubated under hypoxia (CoCl_2_) for 24 h, and then incubated with various concentrations of sorafenib for another 48 h. Both the normoxic and hypoxic cells were suppressed in a dose-dependent manner, but the hypoxic cells were more resistant to sorafenib treatment than the normoxic cells (Fig. 1a). Hypoxic cells possessed a higher level of HIF-2α proteins expression compared to the cells under normoxia which indicated that sorafenib could promote HIF-2α expression while metformin had little effect on the expression of HIF-2α (Fig. 1b). In addition, the combination of metformin and sorafenib inhibited HIF-2α expression to a large degree, indicating that metformin could regain hypoxic cells to be sensitive to sorafenib treatment (Fig. 1c).

### TIP30 was regulated by HIF-2α at protein level under hypoxic conditions in HCC cell lines

**Fig. 2:**
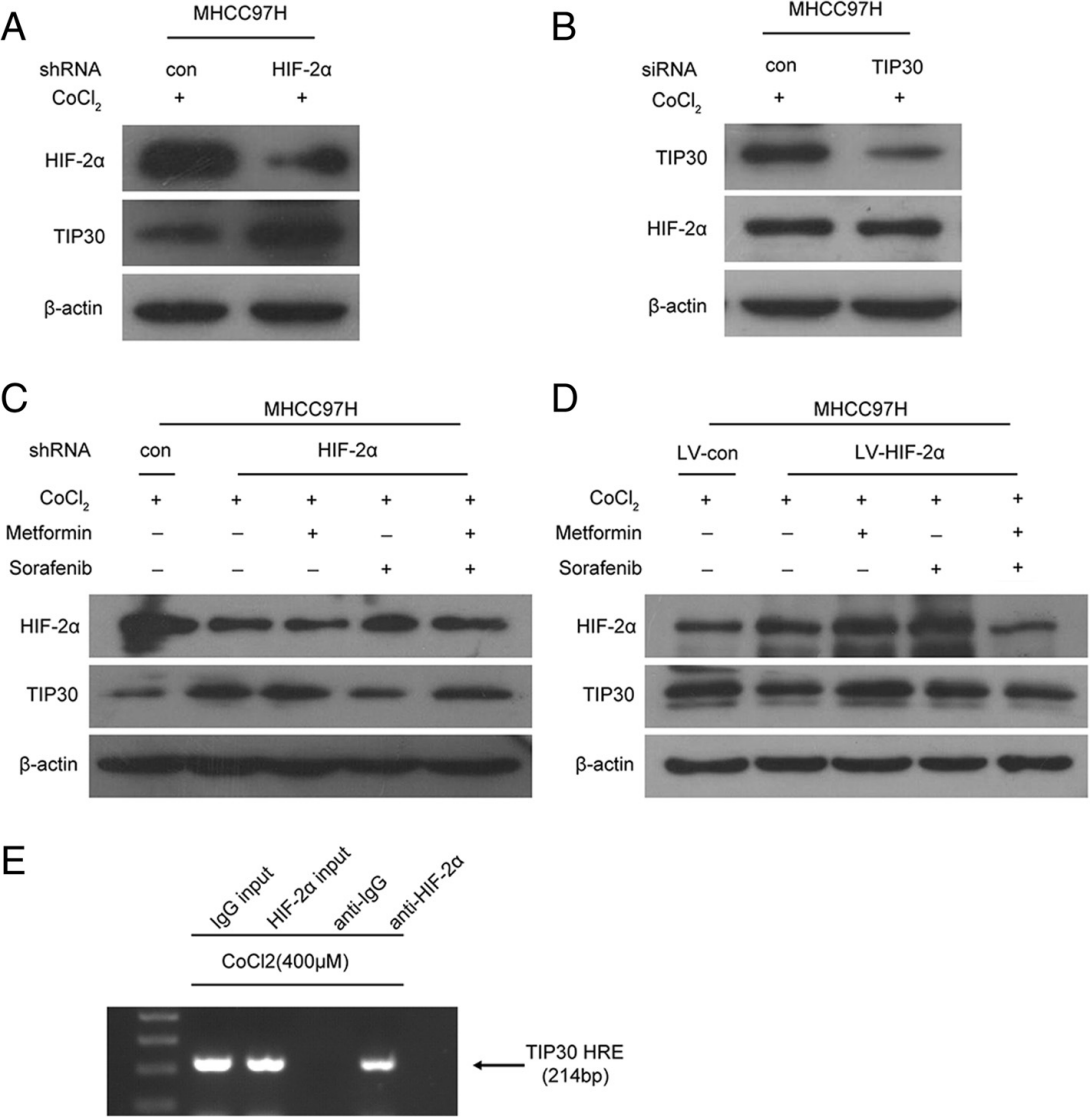
TIP30 was regulated by HIF-2α at protein level in HCC cell lines. Lentivirus infection was used to establish a stable knocking-down or overexpression of HIF-2α in MHCC97H cell lines. The relationship between HIF-2α and TIP30 was detected by Western blot. **a** Knocking-down of HIF-2α upregulated TIP30 expression. **b** Knocking-down of TIP30 did not influence the expression of HIF-2α. **c**, **d** Metformin in combination with sorafenib synergistically inhibited HIF-2α protein expression and subsequently upregulated TIP30 protein expression. **e** MHCC97H cells were exposed to CoCl_2_ (400 μM) for 6 h, and anti-HIF-2α or anti-IgG was used for immunoprecipitation. Immunoprecipitated and purified DNA together with 1 % of input DNA were used for PCR amplification of a 214-bp product encompassing HRE region of TIP30 promoter

Our previous work demonstrated that sorafenib could promote the invasive and metastatic potential of HCC by downregulating the tumor suppressor gene HTATIP2 [15]. MHCC97H cell line with stable knocking-down or overexpression of HIF-2α were established by lentivirus infection. Several articles have suggested that TIP30 knockdown could result in delayed EGFR endocytic degradation and prolonged phosphorylation of AKT (Ser473) and ERK1/2 (Thr202/Tyr204), indicating that downregulation of TIP30 enhances EGFR signaling [24, 25]. Here, we found that knocking-down of HIF-2α could not only upregulate TIP30 expression in MHCC97H and Hep3B cell lines (Fig. 2a, Additional file 1: Figure S1) but also decrease the expression levels of EGFR and its associated downstream molecules, such as phosphorylated extracellular signal-regulated kinases (ERK) and phosphorylated AKT, as determined by Western blot analysis (Additional file 2: Figure S2). However, knocking-down of TIP30 could not cause obvious changes of HIF-2α expression (Fig. 2b). Remarkably, knocking-down of HIF-2α promoted TIP30 expression (Fig. 2c) and overexpression of HIF-2α impaired TIP30 expression (Fig. 2d). Chromatin immunoprecipitation (CHIP) assays were performed to determine whether HIF-2α can bind to the TIP30 promoter, MHCC97H cells were cultured for 6 h at 400 μM CoCl_2_ and fixed with formaldehyde, and then cell nuclei were isolated and sonicated. The sonicated chromatin lysates were immunoprecipitated with anti-IgG or anti-HIF-2α antibodies. Extracted DNA was used to amplify a 214-bp fragment of TIP30 promoter. The amplified 214-bp product was only observed in DNA extracted from hypoxia exposed samples immunoprecipitated with HIF-2α antibody. No product was amplified when nonspecific rabbit anti-IgG was used (Fig. 2e). All these results showed that HIF-2α could bind to the TIP30 promoter under hypoxic conditions.

### Combined metformin and sorafenib therapy suppressed EMT process and promoted apoptosis in hypoxia

**Fig. 3:**
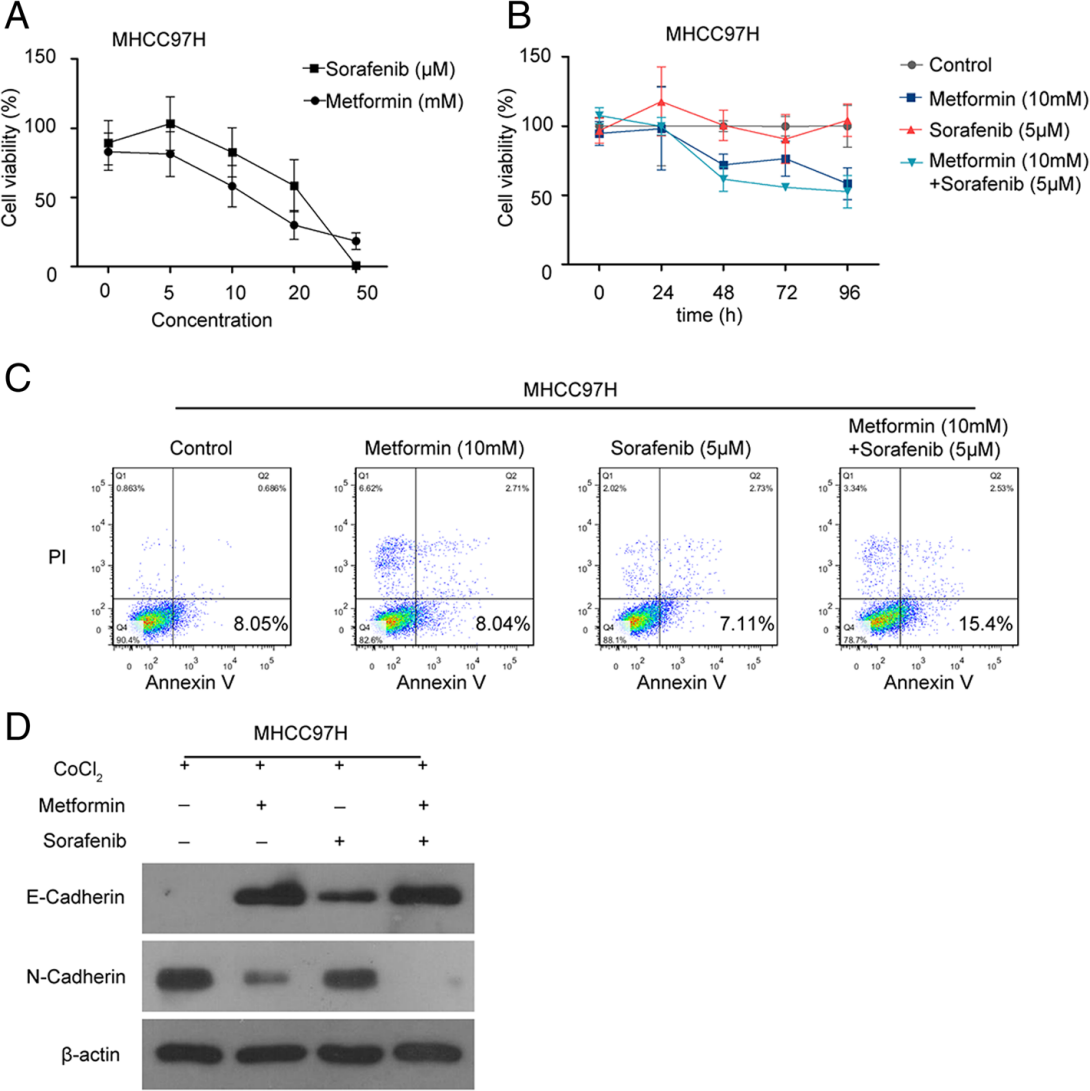
Combined treatment of metformin and sorafenib promoted HCC cells apoptosis and inhibited epithelial-mesenchymal transition process in hypoxia. **a** Cells were cocultured with metformin or sorafenib under cobalt chloride treatment for 48 h. Metformin or sorafenib impaired MHCC97H cell proliferation in a dose-dependent manner. **b** Cells were treated with metformin and sorafenib alone or combined at different time points in the presence of CoCl_2_. Combined treatment inhibited MHCC97H cell proliferation in a time-dependent manner. **c** About 5 × 10^5^ MHCC97H cells were plated in 6-well plates. After treatment with metformin and sorafenib for 48 h in hypoxia, the apoptosis was detected by flow cytometry. Combined therapy significantly promoted MHCC97H cell apoptosis. **d** After treatment with metformin and sorafenib for 48 h in hypoxia, EMT-associated proteins were evaluated by Western blot. Sorafenib alone promoted the process of EMT, while combined treatment inhibited this effect in vitro

To investigate whether metformin could regain tumor cells sensitive to sorafenib, we implemented Cell Counting Kit-8 (CCK8) cell viability assay in MHCC97H cells. The cells were treated with metformin or sorafenib at diverse concentrations for 48 h, and the impact at different time points was evaluated. Metformin or sorafenib monotherapy significantly inhibited the proliferation of MHCC97H cells in a dose- and time-dependent manner, and combination of metformin and sorafenib could further suppress this effect (Fig. 3a, b). We next explored the effects of metformin and sorafenib on tumor cell apoptosis. Flow cytometry analysis showed that metformin or sorafenib alone had no significant effects on tumor cell apoptosis. However, combination of metformin and sorafenib obviously induced apoptosis of MHCC97H cells (Fig. 3c). Our previous work suggested that relatively low dosages of sorafenib promoted HCC invasion and metastasis by downregulating tumor suppressor gene TIP30 [15]. Recent evidence revealed that decreased TIP30 expression could lead to epithelial-mesenchymal transition (EMT), as well as enhance motility and invasion of HCC cells [26]. It has been noted that the EMT may play a major role in tumor invasion and metastasis [27]. These evidence are all in consistent with our results. We concluded that the combination of metformin and sorafenib synergistically impeded EMT and decreased metastasis of HCC (Fig. 3d) which indicated that metformin could enhance the sensitivity of tumor cells to sorafenib.

### Metformin synergized with sorafenib to suppress postoperative intrahepatic recurrence and lung metastasis in orthotopic HCC implantation models

**Fig. 4:**
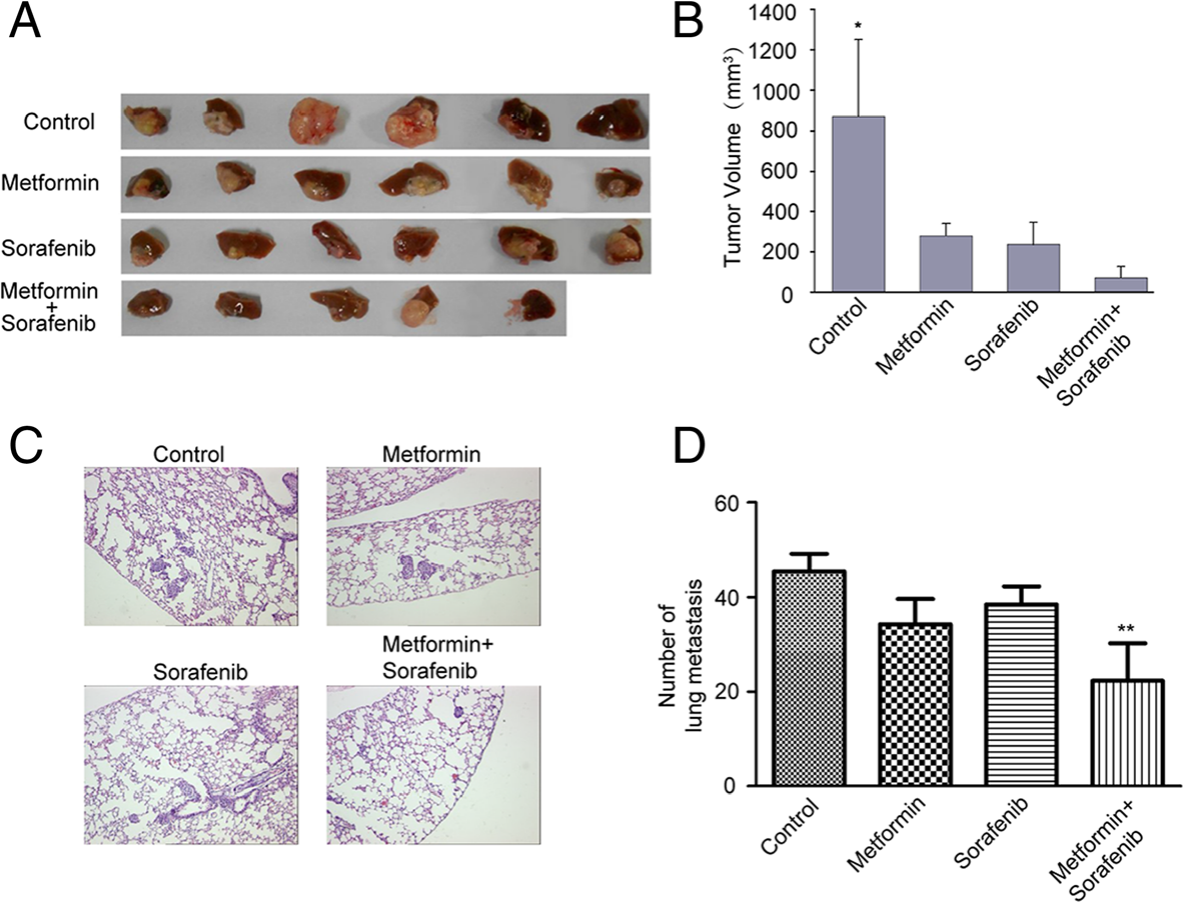
Metformin together with sorafenib suppressed recurrence and metastasis of HCC after surgical resection. **a** Recurrent tumor in different treatment groups. **b** The size (mm^3^) of tumors was determined. **c** Metastatic tumor nodules were detected by HE staining in lung(100×). **d** Average number of metastatic tumor nodules in lung. *Error bars* represent the standard deviation (*P < 0.05, **P < 0.01)

We next examined whether the growth of hepatocellular carcinoma, implanted in their native environment in the liver parenchyma, could also be affected by metformin and sorafenib treatment. Orthotopic tumor xenografts were generated as described in the methods. Fourteen days after the orthotopic implantation, a second operation was carried out to remove the lobe where the tumor was implanted. Then, mice were randomly divided into four groups. On the third day after tumor resection, the mice were orally treated either with 0.9 % sodium chloride (control), 30 mg/kg sorafenib (sorafenib), 200 mg/kg metformin (metformin), or 30 mg/kg sorafenib in combination with 200 mg/kg metformin (sorafenib + metformin) once daily. As shown in Fig. 4a, b, the relapse of vehicle-treated tumors grew remarkably fast 37 days after commencement of treatment. In contrast, the recurrent tumors treated with sorafenib or metformin were significantly (both *P* < 0.05) smaller than vehicle-treated recurrent tumors. The combination of sorafenib and metformin further suppressed recurrent tumor growth, which were much smaller than the vehicle-treated recurrent tumors (*P* < 0.01), and significantly smaller than the recurrent tumors treated with sorafenib (*P* < 0.05) or metformin (*P* < 0.05) monotherapies. Paraffin blocks of 10 % buffered formalin-fixed samples of the lung were prepared, and serial sections were cut at 5 μm and stained with H&E to determine the presence of lung metastases. Metastatic nodules were not significantly reduced in sorafenib-treated mice. However, the number of metastases was remarkably decreased when treated with metformin in combination with sorafenib (Fig. 4c, d).

### Combined therapy inhibited tumor proliferation, angiogenesis and EMT but promoted apoptosis in vivo

**Fig. 5:**
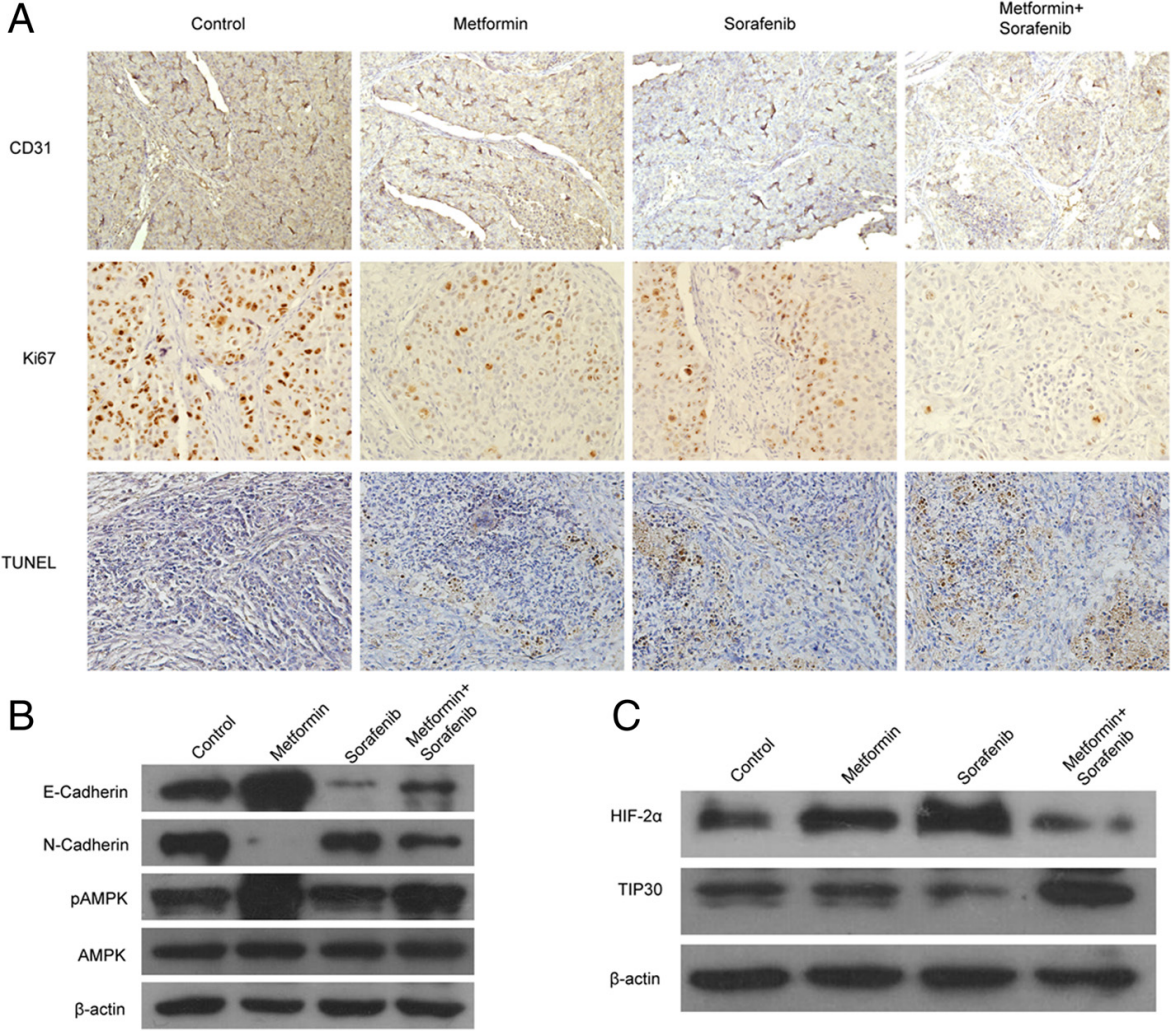
Metformin synergized with sorafenib to suppress angiogenesis, proliferation, and EMT but promote apoptosis in vivo. Representative images of tumor sections were stained with antibody against CD31, Ki67, and TUNEL (magnification ×200). **a** Metformin in combination with sorafenib synergistically minimized both Ki67 and CD31 expression but promoted TUNEL expression. **b** Combined therapy inhibited EMT at protein level which lysated from the tumor tissue, and pAMPK expression was elevated both in metformin group and combined treatment groups. **c** Combined treatment of metformin and sorafenib suppressed HIF-2α protein expression but upregulated TIP30 protein expression evaluated by Western blot

There were fewer Ki67 expression in the metformin or sorafenib treatment groups compared with the control groups, and the combination therapy had even fewer Ki67 positive cells. Metformin or sorafenib alone reduced the CD31 expression, while metformin synergized with sorafenib could further decrease tumor angiogenesis. A small number of TUNEL-positive cells were detected in the control group, whereas plenty of apoptotic tumor cells were detected in metformin or sorafenib monotherapy, the combination therapy resulted in even more TUNEL-positive cells (Fig. 5a). EMT-associated proteins from tumor tissue were detected using Western blot and combined therapy inhibited the process of EMT by upregulating E-cadherin and downregulating N-cadherin protein expression (Fig. 5b). In addition, combined treatment groups inhibited HIF-2α expression but upregulated TIP30 expression at protein level which were consistent with the results in vitro (Fig. 5c).

## Discussion

Surgical operation is the primary treatment for clinical HCC patients. However, recurrence and metastasis are the major factors leading to poor prognosis [4, 5]. Sorafenib is the first-line treatment for advanced hepatocellular carcinoma. However, resistance of tumor cells to sorafenib is one of the main reasons for drug utilization [9]. It seems that finding an effective way to inhibit the resistance may improve the efficiency of sorafenib.

Hypoxia is a prevalent phenomenon in solid tumor in which HIF-2α plays an important role. Previous results indicated that HIF-2α was connected with tumorigenesis, invasion, and metastasis [28, 29]. Here, we revealed that metformin could sensitize hypoxic HCC cells to sorafenib and synergistically suppress the expression of HIF-2α. It has been reported that decreased TIP30 was associated with EMT [26], and our previous results pointed out that sorafenib promoted EMT process by inhibiting TIP30 [15]. In the present study, we found that overexpression of HIF-2α led to downregulation of TIP30 and subsequently promoted the process of EMT, and knocking-down of HIF-2α had the opposite effects. In vitro CHIP assays showed a 214-bp band was amplified immunoprecipitated with anti-HIF-2α antibodies under hypoxic conditions. More broadly, our data support a role for TIP30 as a unique HIF-2α target gene involved in the regulation of cancer recurrence and metastasis.

The previous article reported that exosome-mediated transfer of miR-122 via microRNA (miRNA)-modified adipose tissue-derived mesenchymal stem cells (AMSCs) can enhance the chemosensitivity of HCC cells [30]. Our results showed that combination of metformin and sorafenib inhibited HIF-2α expression to a large degree, indicating that metformin could regain hypoxic HCC cells to be sensitive to sorafenib treatment in vitro. Subsequently, an orthotopic xenograft mouse model was established to explore the influence of metformin combined with sorafenib on recurrence and metastasis after surgical resection of HCC. The subcutaneous tumor was removed and cut into about 2 mm^3^ slice for in situ tumor implantation, and we tried to ensure the uniformity of the tumor as far as possible before implantation. But unavoidably, there may be some disadvantages because the variations may exist in cancer cells. Zhang et al. indicated that arsenic trioxide (As_2_O_3_) induced HCC cancer stem cells (CSCs) differentiation, inhibited recurrence, and prolonged survival after hepatectomy by targeting GLI1 expression [31]. Here, we found that metformin in combination with sorafenib could significantly inhibit the recurrence and metastasis of primary liver cancer in mice after surgical resection. Lower dosage of sorafenib have been found to promote invasion and metastasis of HCC cells [15], and we showed that combined therapy could obviously reduce this effect.

In general, the above features of metformin and the encouraging results presented herein warrant future investigation of the use of metformin for combating HCC, especially in combination with sorafenib.

## Conclusions

Our data indicate that a low dose of sorafenib increases the expression of HIF-2α which downregulated the expression of TIP30 and then promotes HCC invasion and metastasis. Metformin may potentially act as an enhancer of sorafenib to inhibit HCC recurrence and metastasis after surgical resection.

## Methods

### Cell culture and drugs

The highly metastatic human hepatocellular carcinoma cell line MHCC97H obtained from at the Liver Cancer Institute of Fudan University was maintained in Dulbecco’s modified eagle’s medium (DMEM, Gibco, UK), supplemented with 10 % fetal bovine serum, 100 units/ml penicillin, and 100 mg/ml streptomycin and cultured in a 37 °C incubator with 5 % CO_2_ in the air. Sorafenib (Bayer Healthcare, Leverkusen, Germany) was dissolved in dimethyl sulfoxide at a final concentration of 20 mM, and metformin (Bristol-Myers Squibb, China) was dissolved in PBS at a final concentration of 1 M for in vitro assay. Small hairpin RNA (ShRNA) construct against HIF2α (Cat. No. HSH004903-LVRH1GP) and control (Cat. No. CSHCTR001-LVRH1GP), lentiviral plasmid overexpress HIF-2α (Cat. No. EX-M0910-Lv105-5), overexpress control (Cat. No. EX-eGFP-Lv-105), and Lenti-Pac™HIV Expression Packaging Kit (Cat. No. HPK-LvTR-20) were all purchased from GeneCopoeia (America).

### Western blot analysis

Cells were lysed with the lysate containing 25 mM Tris-HCl (pH 7.6), 150 mM NaCl, 1 mM EDTA, 1 % Na-deoxycholate, 0.1 % sodium dodecyl sulfate (SDS), and 1 % Triton X-100, as well as protease and phosphatase inhibitors. Protein concentration was measured with Bradford reagent (Sangon Biotech, Cat. No. B724DB0009). At least 20 μg of sample was used for detecting the protein expression. The antibodies used were as follows: anti-HIF-2 alpha/EPAS1 rabbit polyclonal antibody (1:1000, Novus, Cat. No. NB100-122), anti-TIP30 rabbit monoclonal antibody (1:1000, abcam, Cat. No. ab177961), anti-E-Cadherin rabbit polyclonal antibody (1:1000, abcam, Cat. No. ab15148), and anti-N-Cadherin rabbit polyclonal antibody (1:1000, abcam, Cat. No. ab18203).

### Animals and in vivo experiment

Male BALB/c nude mice weighed 18–20 g and aged 4–6 weeks were purchased from the Animal Research Center, Beijing, China. To construct a nude subcutaneous tumor model, 1 × 10^7^ MHCC97H cells were resuspended in 0.2 ml PBS and injected into the left flank of the mouse. When the tumor volume reached about 1 cm in diameter, the subcutaneous tumor was removed and cut into about 2 mm^3^ slice, and the pieces from one tumor of one mouse were reimplanted into all the subsequent mice. The recipient mice were anesthetized with pentobarbital sodium salt (1 %, 25 mg/kg), and a small piece of tumor was implanted into the left lobe of the liver. Fourteen days after the orthotopic implantation, a second operation was carried out to remove the lobe where the tumor was implanted. Then, the mice were randomly divided into four groups. On the third day, after tumor resection, the mice were orally treated either with 0.9 % sodium chloride (control), 30 mg/kg sorafenib (sorafenib) [15], 200 mg/kg metformin (metformin) [32], or 30 mg/kg sorafenib in combination with 200 mg/kg metformin (sorafenib + metformin) once daily. All the drugs were dissolved in 0.9 % sodium chloride. The mice were treated for 37 days and killed 48 h after the last treatment. The tumor volume (*V*) was measured with vernier caliper and calculated with *V* = 1/2(length × width^2^). The lung tissues were fixed with 10 % formaldehyde, and the HCC tumor tissues were cut into two parts for subsequent fixation with 10 % formaldehyde or frozen resection.

### Immunohistochemistry

The tumor tissue samples and lung tissues were fixed by 10 % formalin, embedded with paraffin, and cut into 5-mm-thick sections. In order to observe the metastatic node in the lung, the lung tissues were stained with hematoxylin and eosin. For immunohistochemistry, the tumor tissue sections were deparaffinized, rehydrated, subsequently subjected to antigen retrieval with 121 °C for 5 min, and incubated with 3 % H_2_O_2_ for 10 min to inactivate endogenous peroxidase. The specimens were blocked with 10 % goat serum for 1 h, and then incubated with primary antibody of Ki67 or CD31 overnight in 4 °C. The next day, the slice was incubated with secondary antibody for 1 h at room temperature. Then, the slice was colored by DAB Substrate-Chromogen System. TUNEL was detected using the TUNEL Apoptosis Detection Kit (KeyGENBioTECH, China, Cat. No. KGA7022).

### Cell viability and apoptosis assay

MHCC97H cells were plated in 96-well plates at 4000 cells/well (*n* = 6) containing 100 μl of DMEM +10 % FBS treated with 400 μM CoCl_2_ and cultured for 24 h, then incubated with sorafenib or metformin for another 48 h. DMEM (100 μl) and CCK8 (10 μl) were added to each well and incubated for 2 h. Then, the absorbance was detected with a microplate reader at a test wavelength of 450 nm.

Annexin V/PI was applied to investigate the impact of sorafenib or metformin on cell apoptosis. After treatments, the cells were added with 5 μl Annexin V and 10 μl PI staining supplied by the Annexin V-FITC Apoptosis Detection Kit (SANGON, Cat. No. BS6336), then the results were measured by flow cytometry using a FACS flow cytometer (Becton Dickinson verse, San Jose, CA).

### Chromatin immunoprecipitation assay

MHCC97H cells were fixed at 37 °C for 10 min with 1 % formaldehyde. The cells were then collected and lysed on ice for 30 min in cell lysis buffer (50 mM EDTA, 1 % SDS, 50 mM Tris-HCl) containing protease inhibitors and 1 mM PMSF. Nuclear chromatin was broken into small fragments by sonicating the nuclear lysate on ice using a Misonix Sonicator 3000 equipped with a microtip. One percent of the volume of samples (input) was saved for the subsequent PCR analysis. The samples were precleared with protein A Sepharose (GE Healthcare, Cat. No. 10043746). Equal aliquots of precleared chromatin samples were incubated overnight at 4 °C with either specific rabbit HIF-2α or nonspecific rabbit antiserum IgG. Immune complexes were collected by incubation with 20 ml protein A Sepharose at 4 °C for 4 h. The complexes were washed once with buffer I (1 % Triton X-100, 2 mM EDTA, 150 mM NaCl, 20 mM Tris-HCl, 0.1 % SDS), buffer II (1 % Triton X-100,2 mM EDTA, 50 mM NaCl, 20 mM Tris-HCl, 0.1 % SDS), buffer III (0.23 mM LiCl, 1 % NP40, 1 % deoxycholate, 1 mM EDTA, 10 mM Tris-HCl), and TE buffer (10 mM Tris-HCl (pH = 8.0),1 mM EDTA), respectively. Immune complexes were then eluted from the beads by incubation twice under agitation at 37 °C with 100 μl of elution buffer (0.1 M NaHCO_3_, 1 % SDS). The eluted material and input was cross-linked at 65 °C for 6 h. Immunoprecipitated DNA was purified by PCR purification kits. Primers used for PCR correspond to the TIP30 promoter region, primers: 5′ primer: 5′-CAAACTTAGGAAGGG TCGCG-3′; 3′ primer: 5′-ATCAGAGCATCCCACCTTCC-3′.

### Statistical analysis

All data were expressed as mean ± SD. The Student *t* test was used for the comparison of measurable variants of the two groups. *P* < 0.05 was defined as statistically significant. All statistical analyses were done using statistical software (SPSS 13.0 for Windows; SPSS, Inc., Chicago, IL).

## Additional files

Additional file 1: TIP30 was regulated by HIF-2α at protein level in Hep3B. (DOCX 66.9 kb) Additional file 2: Knocking-down of HIF-2α decreased the expression levels of EGFR and its associated downstream molecules by upregulating TIP30 expression. (DOCX 55.5 kb)

## Abbreviations

CCK8: Cell Counting Kit-8
CHIP: chromatin immunoprecipitation
EMT: epithelial-mesenchymal transition
HCC: hepatocellular carcinoma
HIF-2α: hypoxia-inducible factors-2α
PDGFR: platelet-derived growth factor receptor
TIP30: 30-kDa HIV Tat-interacting protein (also called CC3 or HTATIP2)
VEGFR-2: vascular endothelial growth factor receptor-2
