# Supplementary material for: Metformin sensitizes sorafenib to inhibit postoperative recurrence and metastasis of hepatocellular carcinoma in orthotopic mouse models
Source: J Hematol Oncol. 2016 Mar 8;9:20. doi: 10.1186/s13045-016-0253-6 (PMC4784359; doi:10.1186/s13045-016-0253-6)
Supplement: Additional file 1: — TIP30 was regulated by HIF-2α at protein level in Hep3B. (DOCX 66.9 kb) [file 13045_2016_253_MOESM1_ESM.docx]

**
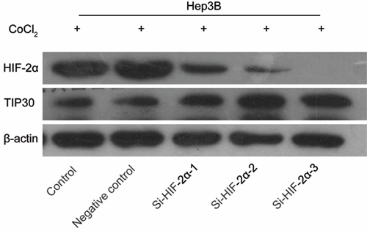
**

**Figure S1**. **TIP30 was regulated by HIF-2α at protein level in Hep3B.** Knocking down of HIF-2α upregulated TIP30 expression
