# Supplementary material for: Metformin sensitizes sorafenib to inhibit postoperative recurrence and metastasis of hepatocellular carcinoma in orthotopic mouse models
Source: J Hematol Oncol. 2016 Mar 8;9:20. doi: 10.1186/s13045-016-0253-6 (PMC4784359; doi:10.1186/s13045-016-0253-6)
Supplement: Additional file 2: — Knocking-down of HIF-2α decreased the expression levels of EGFR and its associated downstream molecules by upregulating TIP30 expression. (DOCX 55.5 kb) [file 13045_2016_253_MOESM2_ESM.docx]

**
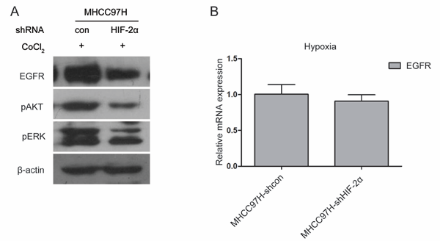
**

**Figure S2**. **Knocking-down of HIF-2****α decreased the expression levels of EGFR and its associated downstream molecules by upregulating TIP30 expression.** (**a**)Western blot analysis of MHCC97H cells with shRNA-control or HIF-2α knockdown. (**b**) Relative mRNA levels determined by real-time RT-PCR are shown for EGFR gene.
